# Special Issue “Saffron (*Crocus sativus*, L.): Omics and Other Techniques in Authenticity, Quality, and Bioactivity Studies”

**DOI:** 10.3390/molecules22010010

**Published:** 2016-12-23

**Authors:** Maria Tsimidou, Petros A. Tarantilis

**Affiliations:** 1 Laboratory of Food Chemistry and Technology, School of Chemistry, Faculty of Sciences, Aristotle University of Thessaloniki, Thessaloniki 54124, Greece; 2 Laboratory of Chemistry, Department of Food Science & Human Nutrition, School of Food, Biotechnology and Development, Agricultural University of Athens, Athens 11855, Greece; ptara@aua.gr

Saffron spice is derived from the dried red stigmas of the *Crocus sativus*, L. flower. Nowadays it is cultivated in various parts of the world such as Iran, India, Greece, Spain, Italy, Morocco, etc. Due to its chemical composition, it is one of the few spices that combines color, taste, and aroma. Rare apocarotenoids, crocetin sugar esters (crocins), picrocrocin, and safranal, respectively, are responsible for these organoleptic properties. These secondary metabolites are found in abundance in the final edible product, i.e., they account for 50% *w*/*w* of the dried stigmas. Their concentration in the stigmas in turn determines their commercial quality.

Saffron is considered as one of the most expensive spices worldwide. Because of its high price and the production constraints, saffron is often a target for various types of adulteration. Nowadays, the addition of artificial colourants and plant material are a common form of fraud. The quality control of saffron typically involves only the determination and quantification of certain colourants suspected as potential adulterants.

Aside from its main use as a spice, saffron exhibits significant biological activity. Its secondary metabolites can alleviate or prevent various health issues such as gastric disorders, cardiovascular disease, insulin resistance, depression, premenstrual syndrome, insomnia, and anxiety. Additionally, saffron presents interesting results in the prevention and maintenance of cancer due to its antioxidant properties.

Last but not least, new products containing saffron have recently appeared in the market. These products deserve the attention of scientists, to determine their quality and properties claimed on their labels.

This Special Issue on saffron focuses on new analytical and molecular approaches that can assure saffron authenticity, determine quality issues, and also highlights the latest trends in bioaccessibility and bioactivity studies.

Concerning saffron authenticity, two papers were included in the issue. The first involves the application of the PCR technique [1]. The authors managed to isolate DNA from pure saffron samples, adulterated samples, and plant material used as an adulterant. From the recovered DNA, newer markers were developed to recognize saffron in mixtures with very low percentages of adulterants. The second paper [2] examines the saffron proteome by gel-electrophoresis and compares it with the proteome of two plant species used as adulterants, *Carthamus tinctorius* and *Gardenia jasminoides*. The protein pattern of saffron was quite distinct from those of the two adulterants, indicating that proteomic analyses can be exploited for detecting possible fraud.

On the topic for saffron quality, the published work in [3] was devoted to the examination of commercial saffron samples using Proton Nuclear Resonance Spectroscopy (^1^H-NMR) and Fourier Transform Infrared Spectroscopy (FT-IR) metabolomics and addressed the difficulties in tracing back the “age” of commercial saffron samples of unknown history, set a limit value above which these products can be considered substandard, and offered a useful tool to combat saffron mislabeling and fraud with low-quality saffron material.

Concerning the biological activity of saffron, four papers were published. The first one studied the decrease of the risk of diabetic cataracts [4]. Crocins were administered to diabetic rats for an 8-week period. The animals were then sacrificed and the parameters involved in the cataract formation were measured in the animal lenses. Crocins showed a powerful inhibitory effect on α-crystallin glycation which resulted in the prevention of diabetic cataracts. The second paper [5] examined whether and to what extend the bioaccessibility of crocins is affected by the presence of strong water-soluble antioxidants, ingredients of the herbs found in commercial tea blends with saffron. The findings suggested that strong radical scavengers may protect crocins from oxidation during digestion. In the third paper, the effect of crocins and crocetin in rat genetic hypertension was studied [6]. The authors concluded that crocetin has endothelium-dependent proleraxin actions, while crocins have procontractile actions that take place via smooth muscle cell mechanisms. Crocetin and crocins activate different mechanisms involved in the vasoconstriction pathway in hypertension. The last published paper in this Special Issue is a review article concerning saffron constituents, which are used for the treatment of anxiety and schizophrenia, and highlights their advantages over currently used anxiolytics and neuroleptics [7].

Finally, we want to express our gratitude to the authors, reviewers, and to the editorial team of Molecules that contributed to the success of this Special Issue on saffron.
